# Injectable Piezoelectric Hydrogel for Vital Pulp Therapy

**DOI:** 10.3390/jfb16120452

**Published:** 2025-12-05

**Authors:** Varun Solanki, Carolina Montoya, Prasanna Neelakantan, Maobin Yang, Santiago Orrego

**Affiliations:** 1 Department of Endodontology, Kornberg School of Dentistry, Temple University, Philadelphia, PA 19140, USA; vsolanki@temple.edu (V.S.); maobin.yang@temple.edu (M.Y.); 2 Department of Oral Health Sciences, Kornberg School of Dentistry, Temple University, Philadelphia, PA 19140, USA; cmontoya@temple.edu; 3 Mike Petryk School of Dentistry, Faculty of Medicine & Dentistry, University of Alberta, Edmonton, AB T6G 2R3, Canada; neelaka1@ualberta.ca; 4 Medical Microbiology and Immunology, University of Alberta, Edmonton, AB T6G 2R3, Canada; 5 Li Ka Shing Institute of Virology, University of Alberta, Edmonton, AB T6G 2R3, Canada; 6 Women & Children’s Health Research Institute, University of Alberta, Edmonton, AB T6G 2R3, Canada; 7 Cancer Research Institute of Northern Alberta, University of Alberta, Edmonton, AB T6G 2R3, Canada; 8 Bioengineering Department, College of Engineering, Temple University, Philadelphia, PA 19122, USA

**Keywords:** piezoelectric hydrogel, pulp capping, barium titanate, odontogenic differentiation, hDPSCs, regenerative dentistry, mechanical stimulation

## Abstract

Vital pulp therapy (VPT) seeks to preserve pulp vitality by using biocompatible with regenerative potential. This study tested the hypothesis that an injectable gelatin methacryloyl (GelMA) hydrogel containing piezoelectric barium titanate promotes odontogenic differentiation of dental pulp stem cells (DPSC) significantly better than a commercially available tricalcium silicate material used for vital pulp therapy. First, the light-curable, injectable piezoelectric hydrogel was engineered and characterized for its physicomechanical, piezoelectric properties and biocompatibility to DPSCs. The effect of this gel on the odontogenic differentiation of DPSCs was determined by measuring the expression level of key genes, compared to Biodentine XP. The hydrogel exhibited excellent injectability (<1 kgf of force), mechanical stability, and generated physiologically relevant voltages under cyclic loading mimicking mastication. MTT and ROS assays show no cytotoxic or damaging oxidative stress effects. When DPSCs were cultured over the materials under cyclic loading, the piezoelectric hydrogel significantly enhanced cell viability and upregulated *COL1A1*, *DSPP*, and *DMP1* expression compared to Biodentine XP and non-piezoelectric hydrogel controls. These findings establish piezoelectric hydrogel as a self-powered, bioactive platform that converts physiological forces into regenerative bioelectric cues, offering a promising next-generation material for vital pulp therapy.

## 1. Introduction

Preserving the vitality of the dental pulp remains the foundation of pulp regeneration since the dentin-pulp complex provides essential sensory, defensive, and reparative tooth functions [1]. Vital pulp therapy (VPT), with appropriate case selection, has emerged as a minimally invasive alternative to conventional root canal therapy, aiming to maintain pulp viability and stimulate reparative dentinogenesis [1]. Conventional pulp capping agents, such as calcium hydroxide and mineral trioxide aggregate (MTA), have demonstrated the ability to form reparative hard tissue (e.g., dentin bridge formation) with significant limitations, including poor mechanical stability, dissolution, tooth discoloration, long setting times, and inadequate modulation of inflammation [1,2,3,4]. Calcium silicate-based cements have shown improvement in material handling and bioactivity, but their antibacterial effects are insufficient against polymicrobial biofilms frequently associated with pulpitis [5,6]. These pulp capping formulations are considered passive since they release ions without actively engaging the biological or mechanical microenvironment of the pulp [7,8].

Multifunctional scaffolds that actively modulate microenvironmental cues to dictate stem cells to promote tissue regeneration are a paradigm from passive biomaterials used in VPT [9,10,11,12,13]. Such biomaterials are designed to integrate antibacterial, immunomodulatory, antioxidant, and/or regenerative functions that dynamically adapt to the hostile pulp microenvironment. Hydrogels, particularly injectable gelatin methacryloyl (GelMA)-based systems, have emerged as versatile scaffolds due to their biocompatibility, injectability, and ability to mimic the extracellular matrix (carrier or vehicle) while incorporating therapeutic agents [2,10,13,14]. Functionalization of GelMA hydrogels with bioactive agents, antimicrobial peptides, or extracellular matrix derivatives has demonstrated enhanced antibacterial activity, immunomodulation, and promotion of odontogenic differentiation in vitro and in vivo [5,15,16,17,18]. Furthermore, environmentally stimuli-responsive hydrogels offer the possibility of on-demand therapeutic release, by responding to local cues such as pH, enzymes, or reactive oxygen species (ROS), thus tailoring treatment to the dynamic pulp microenvironment [9,10,14,19]. Despite these advances, challenges persist in designing biomaterials that simultaneously provide antibacterial protection and promote dentin–pulp regeneration [20].

Piezoelectric dental biomaterials, capable of generating electrical signals in response to mechanical stress (e.g., masticatory force or chewing), represent a novel strategy to harness the physiological forces to produce bioelectric signals capable of eliciting therapeutic effects including stem cell proliferation differentiation and antimicrobial effects [21,22]. In our recent studies, we developed different multifunctional/bioresponsive piezoelectric dental materials including restorative resin composites with antimicrobial and remineralizing effects, prosthodontic dentures with antifungal properties, and piezoelectric hydrogels for antibacterial therapy and bone tissue regeneration for periodontal applications [23,24,25]. These systems demonstrated that mechanically induced electrical charges can inhibit biofilm formation, stimulate osteogenic activity, and promote mineral deposition. Building on this foundation, the present study is the first to explore the use of a piezoelectric hydrogel as a pulp capping material, evaluating its ability to actively modulate the pulp microenvironment and promote dentin tissue regeneration under physiologic mechanical loading. This study introduces an injectable GelMA hydrogel functionalized with barium titanate (BTO) piezoelectric nanoparticles. We hypothesize that the piezoelectric properties of this hydrogel will enhance the cell viability and odontogenic differentiation of human dental pulp stem cells (hDPSCs) subjected to cyclic mechanical loading (electrical charge generation), providing regenerative advantages over conventional pulp capping agents.

## 2. Materials and Methods

### 2.1. Fabrication of Piezoelectric Hydrogels

**Figure 1:**
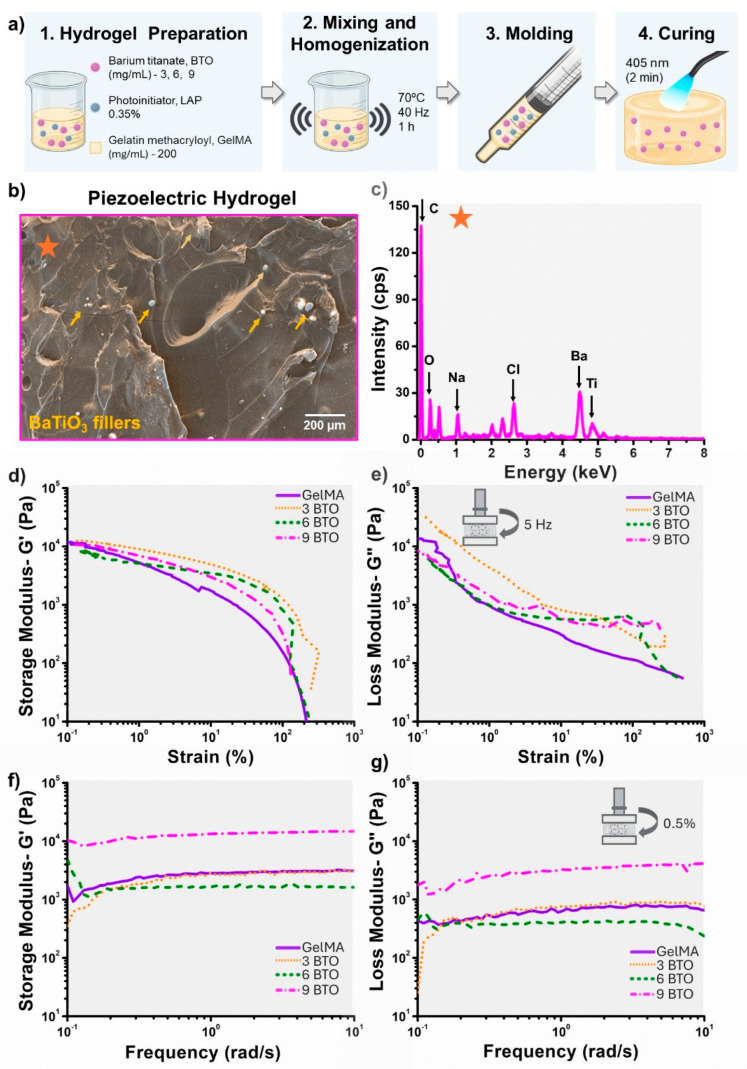
Characterization of piezoelectric hydrogels. (**a**) Schematic illustration of the hydrogel fabrication process and sample preparation. (**b**) Surface micrograph of a piezoelectric hydrogel containing 9 mg/mL of filler. Yellow arrows indicate piezoelectric particles. (**c**) EDS spectrum of a piezoelectric hydrogel with 9 mg/mL filler content, displaying characteristic barium and titanium peaks from the BTO. The spot indicated in (**b**) marks the site of spectrum acquisition. (**d**) Storage modulus and (**e**) loss modulus of cured hydrogels as a function of strain. (**f**) Storage modulus and (**g**) loss modulus of cured hydrogels as a function of strain.

Piezoelectric hydrogels were fabricated by mixing 200 mg/mL of GelMA (Advanced BioMatrix–5208, Carlsbad, CA, USA), BTO nanoparticles (200 nm, US Nanomaterials–US3830, Houston, TX, USA), and sterile phosphate-buffered saline (PBS). Hydrogels with varying BTO concentrations were prepared, including 3 mg/mL, 6 mg/mL, and 9 mg/mL. The photoinitiator lithium phenyl-2,4,6-trimethylbenzoyl phosphinate (LAP, Advanced BioMatrix 5269, Carlsbad, CA, USA) was incorporated at 0.35 wt% of the solubilized GelMA. The lyophilized material was dissolved in PBS (70 °C) using a vortex mixer (Daigger Vortex-Genie, Daigger & Co., Inc., Vernon Hills, IL, USA) until complete solubilization. Later, the BTO particles were introduced while maintaining continuous mixing. Separately, the LAP was dissolved in 100 μL of warm PBS (70 °C) and combined with the GelMA/BTO solution. The mixture was then loaded into a 6 mL polypropylene syringe that served as the mold (Figure 1a). For curing, a 405 nm light source was placed 1 cm from the sample and irradiated for 2 min (total radiant exposure: 24.5 J/cm^2^). After curing, hydrogels were demolded, cut into 12 mm × 6 mm cylinders, and soaked in DI water at 4 °C for 24 h to remove unreacted monomers. As controls, hydrogels without the BTO filler (GelMA only) and Biodentine XP (Septodont, Lancaster, PA, USA) disks were also prepared following the manufacturer’s instructions. All sample preparation was conducted inside a biological safety cabinet, followed by UV sterilization at 254 nm for 15 min before each test.

### 2.2. Physical, Mechanical, and Electromechanical Characterization of Hydrogels

The piezoelectric hydrogels were characterized by their chemical, physical, mechanical, and electromechanical properties, including morphology, filler distribution, chemical composition, rheology, and injectability. The morphology, filler distribution, and chemical composition were evaluated by scanning electron microscopy (SEM) and energy-dispersive X-ray spectroscopy (EDS). Rheological properties of the hydrogels were evaluated at 37 °C using a StressTech HR rheometer (ATS RheoSystems, Bordentown, NJ, USA) equipped with a parallel plate setup (diameter 25 mm, gap 1 mm) [26,27]. Storage and loss moduli of the cured hydrogels were measured through strain-sweep and frequency-sweep analyses. The strain sweep was performed over a range of 0.01% to 500% strain at 5 Hz, while the frequency sweep covered 0.1 to 100 rad/s at a constant strain of 0.5%. Each test used a 3 s conditioning and sampling time, with 10 points per decade, following standard protocols for clinically relevant hydrogel rheological evaluation [28,29]. Injectability was assessed by loading 500 μL of the hydrogels into a 1 mL disposable syringe fitted with a 20-G1/2 needle, ensuring no air bubbles. The syringe was mounted on a rig attached to a universal testing machine (ElectroForce 5500, TA Instruments, New Castle, DE, USA) [30]. The crosshead applied a constant extrusion rate of 2 mL/h (0.45 mm/s) [30]. The force applied to the plunger throughout the injection process was measured and recorded. The electrical output of the hydrogels was measured by placing thin copper electrodes on the top and bottom surfaces of cured disks. Electrodes were connected to a piezoelectric amplifier (TE Connectivity Piezo Film Lab Amplifier, Berwyn, PA, USA) to record open-circuit voltage [31]. Hydrogels were subjected to cyclic loading using a universal testing machine under sinusoidal conditions (0 N to 3 N, 2 Hz), and voltage generation was recorded during stimulation. This loading condition was chosen to mimic the mechanical stress typically supported by pulp capping materials [32,33].

### 2.3. Biomaterials–Dental Pulp Stem Cells’ Interactions

#### 2.3.1. Cell Culture and Seeding

Human dental pulp stem cells (hDPSCs) (Lonza PT-5025, Cohasset, MN, USA) were cultured in DPSC Basal Medium (Lonza PT-3927, Cohasset, MN, USA) and supplemented with the DPSC SingleQuots kit (Lonza PT-4516, Cohasset, MN, USA), which includes fetal bovine serum, L-Glutamine, Gentamicin-Amphotericin, and ascorbic acid solution. Cells were cultured at 37 °C with 5% CO_2_ in a humidified incubator. The cells were passaged at 80% confluence. Cells at passage three (P3) were used for evaluating the hydrogel’s cytocompatibility (MTT assay), oxidative stress response (ROS assay), cellular viability (AlamarBlue assay) and dentinogenic potential (expression of odontoblast/dentin markers). Prior to testing, cell identity and spindle-shaped morphology were verified using phase contrast microscopy, consistent with mesenchymal stem cell characteristics [34].

#### 2.3.2. Cytocompatibility and Cellular Oxidative Stress Response

The cytocompatibility and cellular oxidative stress responses of the piezoelectric hydrogels were conducted using the MTT (3-[4,5-dimethylthiazol-2-yl]-2,5 diphenyl tetrazolium bromide) assay for mitochondrial enzyme activity [35] and by measuring the reactive oxygen species (ROS) produced by the cells [36,37,38]. hDPSCs in P3 were cultured in advanced Dulbecco’s Modified Eagle Medium (DMEM, Gibco 12491015, Grand Island, NY, USA) supplemented with 10% fetal bovine serum (FBS, ATCC 30-2021, Manassas, VA, USA), 100 U/mL of penicillin, and 100 µg/mL of streptomycin (ATCC 30-2300, Manassas, VA, USA) [39]. Cells were seeded at a density of 50,000 cells/mL and incubated at 37 °C with 5% CO_2_ in a humidified incubator. For testing, cells were cultured for 72 h in sterile well plates.

After incubation, hydrogels were transferred to new wells and re-incubated with 0.5 mg/mL of MTT solution (Thermo Fisher Scientific V13154, Waltham, MA, USA) at 37 °C. Following a 3 h incubation, the MTT solution was replaced with an equal volume of dimethyl sulfoxide (DMSO) (Sigma BP231100, St. Louis, MO, USA) and shaken until all formazan crystals were dissolved. Aliquots (150 μL) of the solution were transferred to a 96-well plate. Absorbance at 540 nm (A_540_) was measured using a plate reader (BioTEK Synergy HTX, Winooski, VT, USA). To estimate the cell viability, the MTT values were normalized by the readings obtained from wells without any biomaterial and containing only cells [40].

To measure ROS-induced cell damage, after the incubation period, the hydrogels with the adherent cells were stained with the CellROX^®^ Green Reagent (Thermo Fisher Scientific C10444, Waltham, MA, USA) to a final concentration of 5 μM and re-incubated at 37 °C for 30 min, according to the manufacturer’s instructions. Samples were rinsed with PBS to eliminate excess dye, and ROS levels were measured with a fluorescence plate reader using 485/535 nm excitation/emission settings (Synergy HTX, BioTek, Winooski, VT, USA). As a positive group, inducing oxidative stress, cells were treated by adding 100 ng/mL of a lipopolysaccharide solution (LPS) (*Escherichia coli* 026:B6) (Thermo Fisher Scientific 00-4976-93, Waltham, MA, USA) before the incubation period [41]. As negative control group, cells were pretreated for 1 h at 37 °C with 50 µM pyrrolidine dithiocarbamate (PDTC) to inhibit superoxide anion-induced NF-κB activation, and then stimulated with a lipopolysaccharide (LPS) solution [42,43]. For each experiment six samples were evaluated (*n* = 6).

### 2.4. Dentin Regeneration In Vitro

To evaluate the dentin regeneration potential of the piezoelectric hydrogel containing 9 mg/mL BTO (9 BTO), cells were seeded at a density of 50,000 cells/cm^2^ in 3 mL of media. The hydrogels were then incubated and subjected to cyclic loading (Cell Scale MechanoCulture TX, Waterloo, ON, Canada) for 15 min, 5 times a day, representing eating periods (Supplementary Materials S1). During the experiment, the compressive load magnitude was adjusted from 0.5 to 3 N, resembling the clinical stress in pulp capping materials [32,33]. As a control group, Biodentine disks were also incubated. The culture medium was replaced every 48 h, and assessments of cell viability and gene expression were conducted on days 5 and 10.

#### 2.4.1. Cell Viability

hDPSCs cell viability was measured using alamarBlue (Invitrogen A50100, Waltham, MA, USA) following the manufacturer’s instructions. After 5 and 10 days of incubation, the medium was removed (*N* = 3 samples per group), and 10% (*v*/*v*) alamarBlue was added. Samples were incubated at 37 °C for an additional 4 h. Subsequently, 100 µL aliquots were transferred to a 96-well plate. Absorbance was measured at 570 nm (A570, oxidized alamarBlue) and 600 nm (A600, reduced alamarBlue) to calculate alamarBlue reduction according to the manufacturer’s instructions. The alamarBlue reduction values were normalized by the readings obtained from wells containing only cells as a baseline measurement [44,45].

#### 2.4.2. Odontogenic Differentiation: RNA Extraction and Gene Expression Assays

RNA was isolated from the hydrogels using the RNeasy Mini Kit (QIAGEN, Germantown, MD, USA) following the manufacturer’s protocol, except that lysis was performed directly on the hydrogel by adding 600 µL RTL buffer supplemented with 1% (*v*/*v*) β-mercaptoethanol, followed by vortexing for 1 min [46]. RNA yield and quality were assessed using a NanoDrop (Thermo Fisher Scientific, Waltham, MA, USA). The cDNA synthesis was performed using the iScript Reverse Transcription Supermix (Bio-Rad, Hercules, CA, USA), following the manufacturer’s protocol. Reverse transcription quantitative polymerase chain reaction (RT-qPCR) was performed by using a Bio-Rad CFX96 thermocycler (Bio-Rad, Hercules, CA, USA) and an iTaq Universal SYBR Green Supermix (Bio-Rad, Hercules, CA, USA) with predesigned primer genes associated with hDPSCs differentiation, including Alkaline Phosphatase (*ALP*), Collagen Type I Alpha 1 (*COL1A1*), Dentin Sialophosphoprotein (*DSPP*), and Dentin Matrix Protein-1 (*DMP1*) [47,48]. *GAPDH* was used as a housekeeping gene [49]. The reverse transcription-polymerase chain reaction (RT-qPCR) was performed at 95 °C for 5 min, followed by 40 cycles of 15 s at 95 °C and 1 min at 58 or 55 °C, depending on the primers used. Gene expression was normalized using the comparative Ct (ΔΔCt) method [50]. For each experimental group and gene primer, four samples (*n* = 4) were evaluated.

### 2.5. Statistical Analysis

All data is presented as box plots. The box corresponds to the 25th and 75th percentiles of the data. The whiskers to the standard deviation. One-way ANOVA (α = 0.05) was used to evaluate statistical differences, followed by Tukey’s post hoc test for multiple comparisons (95% confidence). Statistical analyses were conducted in STATGRAPHICS Centurion XVII, version 16.1.18 (Supplemental Materials S2).

## 3. Results

### 3.1. Piezoelectric Hydrogel Characterization

Piezoelectric fillers were observed to be uniformly dispersed within the GelMA matrix, forming small agglomerates of sizes up to 10 μm (Figure 1b). Elemental analysis confirmed the presence of barium and titanium, consistent with the composition of BaTiO_3_ (BTO), as well as carbon, sodium, and chlorine corresponding to the GelMA hydrogel (Figure 1c). From rheology evaluations, both the storage (G′) (Figure 1d) and loss modulus (G″) (Figure 1e) decreased with increasing strain, indicating a transition from the linear viscoelastic region to nonlinear deformation and eventual network breakdown. Specifically, at low strains (~0.1–1%), all hydrogels with different BTO concentrations show high and relatively similar storage modulus (~10^4^ Pa) (Figure 1d). At higher strains (>10%), the storage modulus drops significantly, indicating yielding of the hydrogel network for all BTO combinations. The loss modulus (G″) of all hydrogel combinations decreases with increasing strain, indicating typical strain-softening behavior. However, samples containing BTO fillers slightly maintain higher loss moduli across the strain range compared to the pure hydrogel, indicating enhanced viscoelastic stability and energy-dissipating capacity imparted by the ceramic fillers. Across the entire frequency range (0.1 to 10 rad/s), the storage modulus (G′) (Figure 1f) remained higher than the loss modulus (G″) (Figure 1g) for all formulations, indicating that the hydrogels exhibited dominantly elastic behavior. Overall, both moduli showed minimal frequency dependence, suggesting a stable crosslinked network with consistent mechanical properties under dynamic conditions. The highest mechanical stiffness was observed for the piezoelectric hydrogel with 9 mg/mL of BTO.

### 3.2. Injectability and Electromechanical Characterization of Piezoelectric Hydrogels

**Figure 2:**
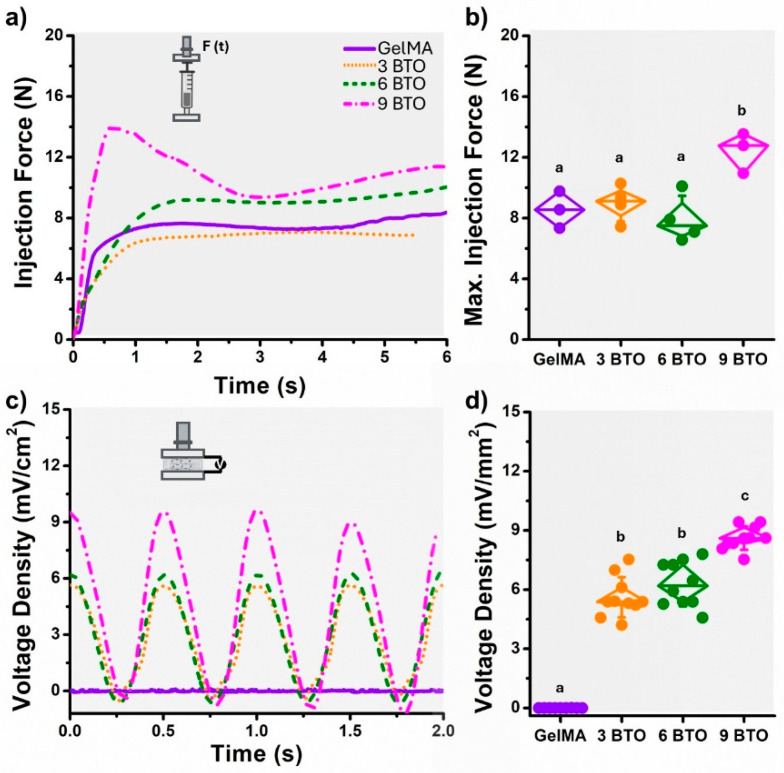
Injectability and electromechanical characterization of piezoelectric hydrogels. (**a**) Time-dependent injection force profiles for hydrogels with 0, 3, 6, and 9 mg/mL filler concentrations. (**b**) Injection force required for the uncured hydrogen to be injected through a 20-gauge needle. All of the measurements were made, simulating a flow rate of 2 mL/h. (**c**) Voltage output of piezoelectric hydrogels under cyclic mechanical loading. The voltage density (mV/cm^2^) was measured as a function of time. (**d**) Maximum voltage per surface area generated after being subjected to a cyclic compression load of 3 N at 2 Hz. Means with different letters are significantly different from each other (*p* ≤ 0.05).

We evaluated the injection force necessary to eject the hydrogels through a syringe connected to a 20-G tip with a flow rate of 2 mL/h mimicking the clinical application of the product. For all hydrogel formulations, the injection measurements (force-time tests) showed an initial linear increase to a peak value, followed by a steady plateau phase (Figure 2a). Overall, we observed that the GelMA (no fillers) and all piezoelectric hydrogels with all BTO concentrations (3 to 9 mg/mL) had an average injection force of 9.3 N (0.95 kgf) (Figure 2b). The slight increase in injection force with higher BTO concentration could be attributed to the increased viscosity, reduced polymer chain mobility, and potential particle agglomeration [27,51]. Nonetheless, the measured forces remain within acceptable clinical limits (<3 kgf), ensuring ergonomic handling comparable to other syringe-based materials [26]. Figure 2c shows the voltage output response of the piezoelectric hydrogels subjected to cyclic mechanical loading (3N, 2Hz). As expected, no voltage was measurable in the GelMA hydrogel since no BTO fillers are included. On the other hand, the piezoelectric hydrogels display cyclic voltage peaks synchronized with the loading frequency, confirming the piezoelectric response due to the addition of BTO. Overall, the voltage density (generated voltage per surface area) was proportional to the amount of BTO (Figure 2d). The highest voltage (9 mV/cm^2^) was measured for the 9 mg/mL BTO concentration.

### 3.3. Cytotoxicity and Oxidative Stress Response of the Piezoelectric Hydrogel

**Figure 3:**
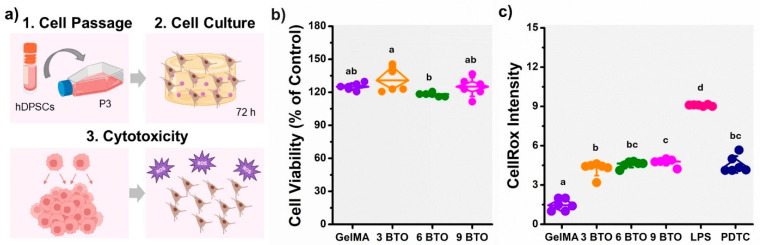
Cytotoxicity and oxidative stress response of piezoelectric hydrogels. (**a**) Schematic representation of the in vitro model used to assess the cytotoxicity and oxidative stress induced by the biomaterials in human dental pulp stem cells (hDPSCs). (**b**) MTT assay showing cell viability after exposure to piezoelectric hydrogels with varying filler concentrations. (**c**) Reactive oxygen species (ROS) production in hDPSCs cultured on different piezoelectric hydrogels. As positive and negative control, cells were treated with lipopolysaccharide (LPS) and pyrrolidine dithiocarbamate (PDTC), respectively. The fluorescence intensity is proportional to the amount of intracellular ROS. The error bars were obtained from *n* = 4 measurements. Means with different letters are significantly different from each other (*p* ≤ 0.05).

The cytotoxicity and oxidative stress response of the piezoelectric hydrogels on hDPSCs are presented in Figure 3. Overall, cell viability remained above 100% for all groups, indicating that none of the hydrogel formulations were cytotoxic (Figure 3b). The 3% BTO group showed slightly enhanced metabolic activity compared to the GelMA control, while 6% and 9% BTO maintained comparable viability levels, confirming positive biocompatibility even at higher filler concentrations, and comparable to that of the GelMA material. These results suggest that piezoelectric composites are not cytotoxic. Assessing intracellular ROS is critical for determining whether hydrogel formulations trigger oxidative stress, which can compromise cell function and biocompatibility. As shown in Figure 3c, an increase in the fluorescence intensity was observed in the piezoelectric hydrogels compared to the control group (GelMA) (*p* < 0.05). Higher fluorescence intensity indicates higher ROS production, oxidative stress in the cells, and inflammation. However, the oxidative stress caused by the piezoelectric hydrogels, especially at the suggested formulations containing <9 mg/mL was significantly lower than seen in the cells treated with LPS (positive control), which is known to activate the inflammatory response significantly [52]. PDTC, a negative control, effectively suppressed ROS as expected. These results indicate that BTO incorporation does not induce oxidative stress, supporting the cytocompatibility of the composite hydrogels.

### 3.4. Odontogenic Potential of the Piezoelectric Hydrogel in Vitro

**Figure 4:**
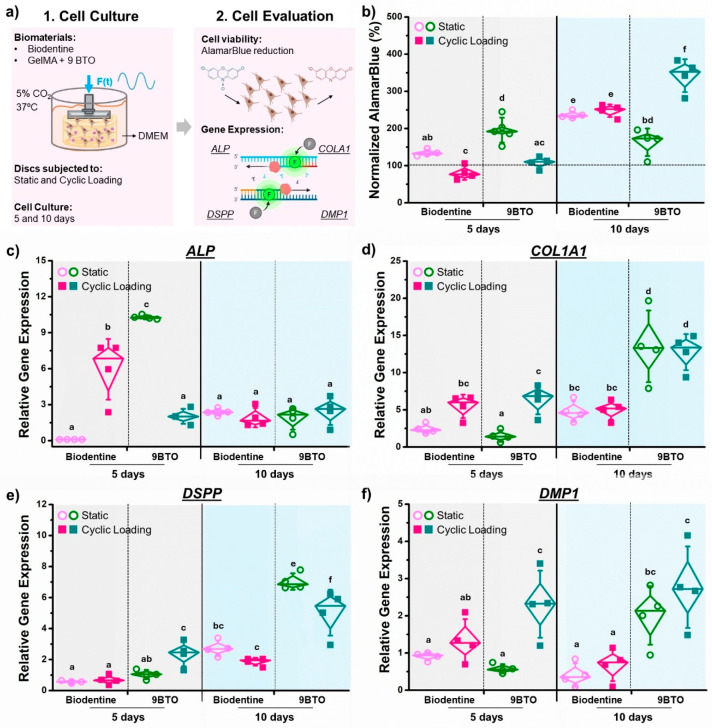
Odontogenic differentiation evaluation of piezoelectric hydrogels in vitro. (**a**) Schematics of the model developed to evaluate odontogenic differentiation of human dental pulp stem cells (hDPSCs) on biomaterials subjected to cyclic mechanical loading and static (no loading). (**b**) Cell viability expressed as alamarBlue reduction (%) and normalized to cells cultured in empty wells. Relative expression of genes associated with the odontogenic differentiation of hDPSCs: (**c**) *ALP* encoding alkaline phosphatase. (**d**) *COLA1* encodes the pro-alpha1 chains of type I collagen. (**e**) *DSPP* encodes the dentin sialophosphoprotein, and (**f**) *DMP1* encoding dentin matrix protein 1. The relative gene expression was normalized to GAPDH (housekeeping). The error bars were obtained from *n* = 4 measurements. Means with different letters are significantly different from each other (*p* ≤ 0.05).

To assess the odontogenic potential of the piezoelectric hydrogel, we examined cell viability and the expression of key regenerative markers (*ALP*, *DSPP*, *COL1A1*, and *DMP1*) in hDPSCs cultured within the hydrogels under static and cyclic loading conditions for 5 and 10 days (Figure 4a). Biodentine XP was included as a control material due to its well-established odontogenic and dentinogenic potential demonstrated in previous studies [53,54]. Overall, the piezoelectric hydrogel (9 BTO) supported hDPSCs viability, with cell metabolic activity increasing over time and further enhanced by cyclic mechanical loading (Figure 4b). At 5 days, hDPSCs cultured on the piezoelectric hydrogel under static conditions showed significantly higher metabolic activity compared to Biodentine, while cyclic loading initially reduced cell viability for both biomaterial groups. By day 10, both tested biomaterials showed increased cell metabolism (>150%). The piezoelectric hydrogel under cyclic loading exhibited the highest metabolic activity (~350%), nearly four times higher than the control. This indicates that mechanical stimulation, through the activation of piezoelectric charges within the hydrogel, enhances cellular proliferation and metabolic function over time. These results underscore the role of piezoelectric activity in sustaining cell metabolism.

To investigate the regeneration potential of piezoelectric hydrogel, we analyzed the expression of early odontogenic differentiation markers, including *ALP* and *COL1A1*. *ALP* regulates the local inorganic phosphate environment necessary for mineral deposition [55]. Elevated *ALP* activity is typically associated with early odontoblast differentiation and initiation of mineralized matrix formation [56]. At 5 days, *ALP* expression was significantly upregulated in the piezoelectric hydrogel under both static and subjected to cyclic loading (Figure 4c). Specifically, Biodentine showed a marked increase (~6–7-fold) under cyclic stimulation, while the piezoelectric hydrogel (9BTO) reached even higher expression levels (~10-fold). The loaded hydrogel showed a 2–3-fold increase in *ALP* expression, lower than its static counterpart. This reduced expression at day 5 likely reflects early odontoblast maturation and progression beyond the initial differentiation stage. By day 10, *ALP* expression decreased across all groups, indicating progression beyond the early differentiation phase. As dentin regeneration progresses, the activity of *ALP* may decrease as odontoblasts turn into quiescent odontoblasts [57]. These findings indicate that cyclic mechanical loading accelerates early odontogenic differentiation, with the BTO phase likely contributing to the rapid activation of *ALP* expression. *COL1A1* encodes the α1 chain of type I collagen, the major organic component of dentin matrix [58]. *COL1A1* upregulation is generally associated with normal dentin formation and function [59]. At 5 days, *COL1A1* expression was upregulated under cyclic loading compared to static conditions for the piezoelectric hydrogel (9BTO) showing the highest increase (~8–10-fold) (Figure 4d). No significant changes were observed in the Biodentine group regardless of the loading condition or incubation time, suggesting that its contribution to remineralization relies more on early differentiation (e.g., *ALP*) rather than strong matrix deposition. By day 10, *COL1A1* expression further increased in the BTO hydrogel, reaching the highest levels (~15–20-fold) under both static and cyclic conditions, surpassing Biodentine’s response (Figure 4d). No significant changes were detected in the Biodentine group under either loading condition. These results indicate that cyclic mechanical stimulation and the presence of BTO promote sustained extracellular matrix gene expression, supporting active dentin matrix formation and tissue maturation.

To assess late-stage odontogenic differentiation, we analyzed the expression of *DSPP* and *DMP1*, key markers associated with odontoblast maturation and dentin matrix mineralization. *DSPP* encodes both dentin sialoprotein (DSP) and dentin phosphoprotein (DPP), which are critical for the development and mineralization of dentin [60]. At 5 days, *DSPP* expression remained low for all groups, with a significant increase observed in the 9BTO hydrogel under cyclic loading (Figure 4e). The early *DSPP* upregulation in 9BTO under mechanical loading suggests accelerated odontoblast maturation and premature activation of dentin matrix genes. By day 10, *DSPP* expression markedly increased in both materials, with the piezoelectric hydrogel under cyclic loading showing the highest levels (fivefold higher than Biodentine). The highest increase was observed for the static 9BTO group, suggesting sustained odontogenic activity driven by the material’s intrinsic piezoelectric and bioactive properties. *DMP1* encodes dentin matrix protein 1, an extracellular matrix protein that regulates osteoblast gene expression and mineralization of bone [61]. At 5 days, *DMP1* expression in the 9BTO hydrogel under cyclic loading was markedly higher (~3-fold) than under static conditions and higher than Biodentine (Figure 4f), indicating early activation of dentin matrix mineralization pathways. By day 10, *DMP1* levels in 9BTO remained significantly elevated under cyclic loading, confirming sustained odontoblastic maturation and matrix secretion. Biodentine showed little expression regardless of the loading condition. These findings suggest that piezoelectric stimulation enhances late-stage odontogenic differentiation and supports prolonged mineralization signaling.

## 4. Discussion

This study presents a light-curable, syringe-injectable piezoelectric hydrogel (GelMA added with BTO fillers) designed and tailored for VPT. The composite hydrogel demonstrated excellent injectability, mechanical resilience, and stable mechanoelectric responsiveness, generating physiologically relevant voltages under cyclic forces that mimic mastication. Importantly, the material was highly cytocompatible and promoted the upregulation of odontogenic and dentin-matrix genes (*ALP*, *COL1A1*, *DSPP*, and *DMP1*) in hDPSCs, particularly under dynamic loading. Compared with Biodentine XP, the piezoelectric hydrogel elicited stronger and more sustained odontogenic responses, indicating that mechanically induced electrical stimulation from the BTO phase accelerates early differentiation and supports late-stage mineralization. These findings support the acceptance of our original hypothesis. Clinically, these findings suggest that normal chewing could act as a natural trigger for continuous, low-level bioelectric activation enhancing reparative dentin formation and positioning piezoelectric hydrogel (GelMA + BTO) as a next-generation smart pulp-capping material capable of translating everyday mechanical function into regenerative signaling.

The enhanced odontogenic differentiation observed in the piezoelectric hydrogel is likely mediated by the localized electric fields and surface charge redistribution generated by BTO during cyclic loading stimulation. These produced microcurrents can modulate ion fluxes, particularly Ca^2+^ and Na^+^, and activate downstream signaling cascades such as ERK1/2, MAPK, and Wnt/β-catenin, which are known to regulate odontoblast differentiation and mineralized tissue formation [62,63,64]. Previous studies have shown that BTO nanoparticles can enhance osteo/odontogenic differentiation through electrochemical polarization, leading to increased expression of mineralization-related genes and upregulation of *ALP*, *COL1A1*, *DSPP*, and *DMP1* [63,64]. In this context, the synergistic effects of GelMA’s biocompatible matrix and the BTO’s electromechanical responsiveness provide a dynamic microenvironment that mimics the native mechanotransductive cues of the dentin–pulp complex. The higher gene expression observed under cyclic loading supports the concept that repeated mechanical activation amplifies bioelectric signaling, accelerating the transition from early to late odontogenic stages. This mechanobiological behavior highlights the potential of piezoelectric hydrogels to function not only as passive scaffolds but as bioactive electromechanical transducers, capable of converting physiological forces into regenerative stimuli.

Our piezoelectric gel integrates well into a chairside VPT workflow. From a handling standpoint, regardless of the BTO content, the injection forces through a 20-gauge needle (~0.9–1.1 kgf), remained comfortably below clinical thresholds, allowing conformal placement into small or irregular exposures prior to light curing at 405 nm in ~2 min. After curing, the gel exhibited an elastic, frequency-stable profile (G′ > G″), maintaining dimensional integrity under early functional loads. In addition, the BTO content is tunable, enabling clinicians to balance stiffness, injectability, and voltage output to meet indication-specific needs—features that address core priorities of speed, precision, and predictability, while also introducing a stimulus-responsive property absent in passive ion-releasing cements.

While Biodentine primarily induced an early *ALP* increase indicative of initial differentiation, it did not maintain high expression of late markers (*COL1A1*, *DSPP*, *DMP1*) over time. In contrast, the self-powered piezoelectric stimulation in the piezoelectric hydrogel enhanced hDPSCs viability and strongly upregulated these late odontogenic genes, demonstrating prolonged activation of matrix synthesis and mineralization pathways. The relatively lower *ALP* expression in the piezoelectric hydrogel at day 5, despite elevated *COL1A1* and later *DSPP*/*DMP1*, suggests an accelerated transition beyond early differentiation toward active matrix organization and mineralization readiness [65,66]. Conversely, Biodentine’s transient *ALP* peak likely reflects ion-release–mediated early cues without a corresponding progression to late odontoblast maturation within the experimental window cues [67,68]. Collectively, these findings indicate that the piezoelectric hydrogel supports a more complete odontogenic trajectory, advancing from early matrix deposition to dentin-specific gene activation more effectively than the conventional calcium silicate cement.

From a translational perspective, the injectable and light-curable GelMA–BTO piezoelectric hydrogel offers several advantages for clinical use in VPT and regenerative endodontics. Its shear-thinning and syringe-extrudable behavior allows precise placement into small or irregular pulp exposures, while rapid photopolymerization ensures on-demand stabilization without excessive heat or chemical irritation. Importantly, the self-powered piezoelectric response enables continuous microelectrical stimulation triggered by natural masticatory forces, eliminating the need for external devices and creating a bioactive environment that promotes reparative dentin formation in situ. Compared with static bioactive cements such as Biodentine, this dynamic stimulation model could enhance treatment outcomes by maintaining long-term cellular activation during the early healing period. The absence of cytotoxicity and low ROS generation observed in vitro also supports the material’s biocompatibility and safety for in vivo applications. Future studies should focus on validating in vivo regenerative performance, long-term interfacial mineralization, and polarization stability to further establish GelMA–BTO as a next-generation smart pulp-capping and dentin–pulp regeneration platform that integrates seamlessly with the body’s natural biomechanics.

We acknowledge that this in vitro model, designed to approximate clinical handling and mastication-like loading, cannot completely reproduce the vascular–immune complexity of the pulp. Future work should incorporate co-culture/organotypic systems to interrogate inflammatory crosstalk and ROS homeostasis under loading [69]; evaluate antibacterial performance against endodontic pathogens and multispecies biofilms under dynamic conditions, given prior evidence that piezoelectric charges modulate oral microbiota [24,25,70], and examine degradation, ion-leaching, and mechanical stability under thermo-mechanical cycling. In vivo studies remain essential to confirm not only the capacity of the material to preserve pulp vitality, but also to assess the quality, continuity, and thickness of the resulting dentin bridge, evaluate the extent of re-innervation and vascular integration within the regenerated tissue, monitor inflammatory resolution, and determine whether the piezoelectric cues translate into meaningful improvements in functional regeneration. Materials refinements (particle size/surface modification, BTO content) may elevate voltage yield without compromising injectability or oxidative-stress profiles. By converting routine mastication into localized bioelectric cues, our piezoelectric hydrogel reframes VPT as an actively responsive rather than passive intervention. Its delivery and rapid light-curing support precise placement in small or irregular exposures, while cyclic loading enhances matrix gene expression (*COL1A1*) and late odontogenic markers (*DSPP*, *DMP1*), which aligns with rapid, high-quality reparative dentin formation. If confirmed in preclinical and clinical studies, this approach could broaden VPT indications and improve early healing trajectories by leveraging everyday function as a therapeutic driver.

## 5. Conclusions

This study developed a light-curable, injectable piezoelectric hydrogel (GelMA + barium titanate) for Vital Pulp Therapy (VPT) that combines excellent injectability (injection force < 1 kgf), smooth syringe delivery due to its shear-thinning behavior, and acceptable biocompatibility with enhanced regenerative potential. Under cyclic mechanical stimulation (similar to the forces experienced during chewing), the piezoelectric hydrogel generated physiologically relevant electrical charges, significantly improving the viability and odontogenic differentiation of human dental pulp stem cells (hDPSCs), as evidenced by upregulated *COL1A1*, *DSPP*, and *DMP1* expression. These results highlight piezoelectric hydrogel as a self-powered, next-generation biomaterial capable of converting masticatory forces into bioelectric signals to promote dentin–pulp regeneration.

## Data Availability

The original contributions presented in the study are included in the article, further inquiries can be directed to the corresponding author.

## Supplementary Materials

The following supporting information can be downloaded at: https://www.mdpi.com/article/10.3390/jfb16120452/s1.
