# Supplementary material for: Injectable Piezoelectric Hydrogel for Vital Pulp Therapy
Source: J Funct Biomater. 2025 Dec 5;16(12):452. doi: 10.3390/jfb16120452 (PMC12733838; doi:10.3390/jfb16120452)
Supplement: Supplementary file 1 [file jfb-16-00452-s001.zip › jfb-3995708-supplementary.pdf]

Supplemental Materials S1: Experimental set-up

Piezoelectric hydrogels were placed on a 6-well plate and submerged in 3 mL of media. Samples were subjected to compression forces using a Cell Scale MechanoCulture TX. (Fig.S1). The forces were applied for 15 min, 5 times a day, representing eating periods.

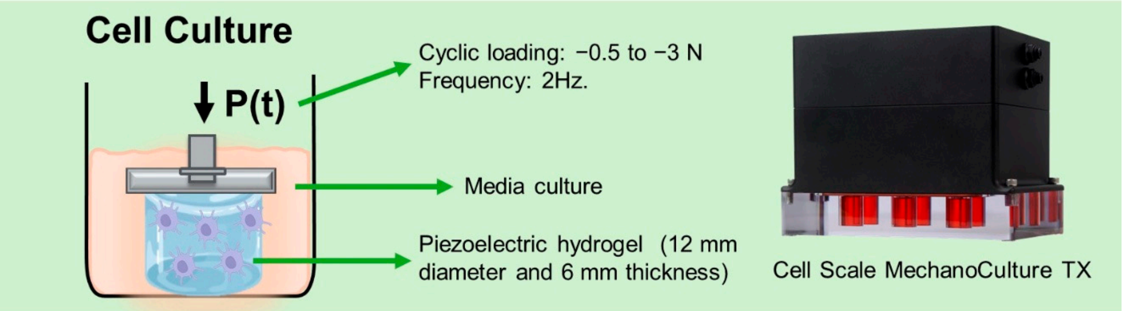

Figure S1. Experimental set-up used to evaluate odontogenic differentiation of human dental pulp stem cells (hDPSCs) on biomaterials subjected to cyclic mechanical loading and static (no loading). a) Scheme of the cyclic loading configuration and b) image of the actuator used for experimentation.

Supplemental Materials S2: Statistical Analysis

- ***Max. Injection Force***

ANOVA Table

Source	*Sum of Squares*	*Df*	*Mean Square*	*F-Ratio*	*P-Value*
Between groups	38.9885	3	12.9962	7.23	0.0073
Within groups	17.9819	10	1.79819		
Total (Corr.)	56.9705	13			

Contrast	*Sig.*	*Difference*	*+/- Limits*
GelMA - 3 BTO		-0.4325	2.28202
GelMA - 6 BTO		0.6375	2.28202
GelMA - 9 BTO	*	-3.87	2.43959
3 BTO - 6 BTO		1.07	2.11274
3 BTO - 9 BTO	*	-3.4375	2.28202
6 BTO - 9 BTO	*	-4.5075	2.28202

* denotes a statistically significant difference.

- ***Voltage Density***

ANOVA Table

Source	*Sum of Squares*	*Df*	*Mean Square*	*F-Ratio*	*P-Value*
Between groups	372.776	3	124.259	180.96	0.0000
Within groups	24.0338	35	0.68668		
Total (Corr.)	396.81	38			

Contrast	*Sig.*	*Difference*	*+/- Limits*
GelMA - 3 BTO	*	-5.60823	0.772952
GelMA - 6 BTO	*	-6.28223	0.772952
GelMA - 9 BTO	*	-8.60823	0.772952
3 BTO - 6 BTO		-0.674	0.752337
3 BTO - 9 BTO	*	-3.0	0.752337

6 BTO - 9 BTO	*	-2.326	0.752337

* denotes a statistically significant difference.

- ***Cell Viability***

ANOVA Table

Source	*Sum of Squares*	*Df*	*Mean Square*	*F-Ratio*	*P-Value*
Between groups	0.0623721	3	0.0207907	3.82	0.0260
Within groups	0.108994	20	0.0054497		
Total (Corr.)	0.171366	23			

Contrast	*Sig.*	*Difference*	*+/- Limits*
GelMA - 3 BTO		-0.0720567	0.0889065
GelMA - 6 BTO		0.0720583	0.0889065
GelMA - 9 BTO		0.00379333	0.0889065
3 BTO - 6 BTO	*	0.144115	0.0889065
3 BTO - 9 BTO		0.07585	0.0889065
6 BTO - 9 BTO		-0.068265	0.0889065

* denotes a statistically significant difference

- ***CellRox Intensity***

ANOVA Table

Source	*Sum of Squares*	*Df*	*Mean Square*	*F-Ratio*	*P-Value*
Between groups	178.294	5	35.6588	208.87	0.0000
Within groups	5.12168	30	0.170723		
Total (Corr.)	183.416	35			

Contrast	*Sig.*	*Difference*	*+/- Limits*
GelMA - 3 BTO	*	-2.76262	0.487191
GelMA - 6 BTO	*	-3.0974	0.487191
GelMA - 9 BTO	*	-3.26273	0.487191
GelMA - LPS	*	-7.595	0.487191
GelMA - PDTC	*	-3.08217	0.487191
3 BTO - 6 BTO		-0.33478	0.487191
3 BTO - 9 BTO	*	-0.500113	0.487191
3 BTO - LPS	*	-4.83238	0.487191
3 BTO - PDTC		-0.319547	0.487191
6 BTO - 9 BTO		-0.165333	0.487191
6 BTO - LPS	*	-4.4976	0.487191
6 BTO - PDTC		0.0152333	0.487191
9 BTO - LPS	*	-4.33227	0.487191
9 BTO - PDTC		0.180567	0.487191
LPS - PDTC	*	4.51283	0.487191

* denotes a statistically significant difference.

- ***Normalized AlamarBLue***

ANOVA Table

Source	*Sum of Squares*	*Df*	*Mean Square*	*F-Ratio*	*P-Value*
Between groups	20.501	7	2.92871	48.41	0.0000
Within groups	1.45207	24	0.0605031		
Total (Corr.)	21.9531	31			

Contrast	*Sig.*	*Difference*	*+/- Limits*
Biodentine - 5 days- Static - Biodentine - 5 days- Loading	*	0.54	0.358974

Biodentine - 5 days- Static - 9 BTO - 5 days- Static	*	-0.49	0.358974
Biodentine - 5 days- Static - 9 BTO - 5 days- Loading		0.2625	0.358974
Biodentine - 5 days- Static - Biodentine - 10 days- Static	*	-1.0225	0.358974
Biodentine - 5 days- Static - Biodentine - 10 days- Loading	*	-1.135	0.358974
Biodentine - 5 days- Static - 9 BTO - 10 days- Static		-0.2875	0.358974
Biodentine - 5 days- Static - 9 BTO - 10 days- Loading	*	-2.08	0.358974
Biodentine - 5 days- Loading - 9 BTO - 5 days- Static	*	-1.03	0.358974
Biodentine - 5 days- Loading - 9 BTO - 5 days- Loading		-0.2775	0.358974
Biodentine - 5 days- Loading - Biodentine - 10 days- Static	*	-1.5625	0.358974
Biodentine - 5 days- Loading - Biodentine - 10 days- Loading	*	-1.675	0.358974
Biodentine - 5 days- Loading - 9 BTO - 10 days- Static	*	-0.8275	0.358974
Biodentine - 5 days- Loading - 9 BTO - 10 days- Loading	*	-2.62	0.358974
9 BTO - 5 days- Static - 9 BTO - 5 days- Loading	*	0.7525	0.358974
9 BTO - 5 days- Static - Biodentine - 10 days- Static	*	-0.5325	0.358974
9 BTO - 5 days- Static - Biodentine - 10 days- Loading	*	-0.645	0.358974
9 BTO - 5 days- Static - 9 BTO - 10 days- Static		0.2025	0.358974
9 BTO - 5 days- Static - 9 BTO - 10 days- Loading	*	-1.59	0.358974
9 BTO - 5 days- Loading - Biodentine - 10 days- Static	*	-1.285	0.358974
9 BTO - 5 days- Loading - Biodentine - 10 days- Loading	*	-1.3975	0.358974
9 BTO - 5 days- Loading - 9 BTO - 10 days- Static	*	-0.55	0.358974
9 BTO - 5 days- Loading - 9 BTO - 10 days- Loading	*	-2.3425	0.358974
Biodentine - 10 days- Static - Biodentine - 10 days- Loading		-0.1125	0.358974
Biodentine - 10 days- Static - 9 BTO - 10 days- Static	*	0.735	0.358974
Biodentine - 10 days- Static - 9 BTO - 10 days- Loading	*	-1.0575	0.358974
Biodentine - 10 days- Loading - 9 BTO - 10 days- Static	*	0.8475	0.358974
Biodentine - 10 days- Loading - 9 BTO - 10 days- Loading	*	-0.945	0.358974
9 BTO - 10 days- Static - 9 BTO - 10 days- Loading	*	-1.7925	0.358974

* denotes a statistically significant difference.

- ***Gene Expression: ALP***

ANOVA Table

Source	*Sum of Squares*	*Df*	*Mean Square*	*F-Ratio*	*P-Value*
Between groups	292.26	7	41.7514	33.84	0.0000
Within groups	29.609	24	1.23371		
Total (Corr.)	321.869	31			

Contrast	*Sig.*	*Difference*	*+/- Limits*
Biodentine - 5 days- Static - Biodentine - 5 days- Loading	*	-5.84478	1.62099
Biodentine - 5 days- Static - 9 BTO - 5 days- Static	*	-10.1866	1.62099
Biodentine - 5 days- Static - 9 BTO - 5 days- Loading	*	-1.92814	1.62099
Biodentine - 5 days- Static - Biodentine - 10 days- Static	*	-2.28191	1.62099
Biodentine - 5 days- Static - Biodentine - 10 days- Loading	*	-1.8183	1.62099
Biodentine - 5 days- Static - 9 BTO - 10 days- Static	*	-1.78093	1.62099
Biodentine - 5 days- Static - 9 BTO - 10 days- Loading	*	-2.37457	1.62099
Biodentine - 5 days- Loading - 9 BTO - 5 days- Static	*	-4.34183	1.62099
Biodentine - 5 days- Loading - 9 BTO - 5 days- Loading	*	3.91663	1.62099
Biodentine - 5 days- Loading - Biodentine - 10 days- Static	*	3.56287	1.62099
Biodentine - 5 days- Loading - Biodentine - 10 days- Loading	*	4.02647	1.62099
Biodentine - 5 days- Loading - 9 BTO - 10 days- Static	*	4.06385	1.62099
Biodentine - 5 days- Loading - 9 BTO - 10 days- Loading	*	3.4702	1.62099
9 BTO - 5 days- Static - 9 BTO - 5 days- Loading	*	8.25846	1.62099
9 BTO - 5 days- Static - Biodentine - 10 days- Static	*	7.9047	1.62099
9 BTO - 5 days- Static - Biodentine - 10 days- Loading	*	8.3683	1.62099
9 BTO - 5 days- Static - 9 BTO - 10 days- Static	*	8.40568	1.62099
9 BTO - 5 days- Static - 9 BTO - 10 days- Loading	*	7.81203	1.62099
9 BTO - 5 days- Loading - Biodentine - 10 days- Static		-0.353768	1.62099

9 BTO - 5 days- Loading - Biodentine - 10 days- Loading		0.109838	1.62099
9 BTO - 5 days- Loading - 9 BTO - 10 days- Static		0.147212	1.62099
9 BTO - 5 days- Loading - 9 BTO - 10 days- Loading		-0.44643	1.62099
Biodentine - 10 days- Static - Biodentine - 10 days- Loading		0.463605	1.62099
Biodentine - 10 days- Static - 9 BTO - 10 days- Static		0.50098	1.62099
Biodentine - 10 days- Static - 9 BTO - 10 days- Loading		-0.0926625	1.62099
Biodentine - 10 days- Loading - 9 BTO - 10 days- Static		0.037375	1.62099
Biodentine - 10 days- Loading - 9 BTO - 10 days- Loading		-0.556267	1.62099
9 BTO - 10 days- Static - 9 BTO - 10 days- Loading		-0.593642	1.62099

* denotes a statistically significant difference.

- ***Gene Expression: COL1A1***

ANOVA Table

Source	*Sum of Squares*	*Df*	*Mean Square*	*F-Ratio*	*P-Value*
Between groups	547.22	7	78.1743	15.53	0.0000
Within groups	120.822	24	5.03427		
Total (Corr.)	668.042	31			

Contrast	*Sig.*	*Difference*	*+/- Limits*
Biodentine - 5 days- Static - Biodentine - 5 days- Loading		-3.0367	3.27448
Biodentine - 5 days- Static - 9 BTO - 5 days- Static		0.958963	3.27448
Biodentine - 5 days- Static - 9 BTO - 5 days- Loading	*	-3.96714	3.27448
Biodentine - 5 days- Static - Biodentine - 10 days- Static		-2.35398	3.27448
Biodentine - 5 days- Static - Biodentine - 10 days- Loading		-2.56265	3.27448
Biodentine - 5 days- Static - 9 BTO - 10 days- Static	*	-11.1056	3.27448
Biodentine - 5 days- Static - 9 BTO - 10 days- Loading	*	-10.3277	3.27448
Biodentine - 5 days- Loading - 9 BTO - 5 days- Static	*	3.99566	3.27448
Biodentine - 5 days- Loading - 9 BTO - 5 days- Loading		-0.93044	3.27448
Biodentine - 5 days- Loading - Biodentine - 10 days- Static		0.682713	3.27448
Biodentine - 5 days- Loading - Biodentine - 10 days- Loading		0.47404	3.27448
Biodentine - 5 days- Loading - 9 BTO - 10 days- Static	*	-8.06889	3.27448
Biodentine - 5 days- Loading - 9 BTO - 10 days- Loading	*	-7.29097	3.27448
9 BTO - 5 days- Static - 9 BTO - 5 days- Loading	*	-4.9261	3.27448
9 BTO - 5 days- Static - Biodentine - 10 days- Static	*	-3.31295	3.27448
9 BTO - 5 days- Static - Biodentine - 10 days- Loading	*	-3.52162	3.27448
9 BTO - 5 days- Static - 9 BTO - 10 days- Static	*	-12.0645	3.27448
9 BTO - 5 days- Static - 9 BTO - 10 days- Loading	*	-11.2866	3.27448
9 BTO - 5 days- Loading - Biodentine - 10 days- Static		1.61315	3.27448
9 BTO - 5 days- Loading - Biodentine - 10 days- Loading		1.40448	3.27448
9 BTO - 5 days- Loading - 9 BTO - 10 days- Static	*	-7.13845	3.27448
9 BTO - 5 days- Loading - 9 BTO - 10 days- Loading	*	-6.36053	3.27448
Biodentine - 10 days- Static - Biodentine - 10 days- Loading		-0.208672	3.27448
Biodentine - 10 days- Static - 9 BTO - 10 days- Static	*	-8.7516	3.27448
Biodentine - 10 days- Static - 9 BTO - 10 days- Loading	*	-7.97369	3.27448
Biodentine - 10 days- Loading - 9 BTO - 10 days- Static	*	-8.54293	3.27448
Biodentine - 10 days- Loading - 9 BTO - 10 days- Loading	*	-7.76501	3.27448
9 BTO - 10 days- Static - 9 BTO - 10 days- Loading		0.777915	3.27448

* denotes a statistically significant difference.

- ***Gene Expression: DSPP***

ANOVA Table

Source	*Sum of Squares*	*Df*	*Mean Square*	*F-Ratio*	*P-Value*
Between groups	145.354	7	20.7648	45.96	0.0000
Within groups	10.8421	24	0.451753		
Total (Corr.)	156.196	31			

Contrast	*Sig.*	*Difference*	*+/- Limits*
Biodentine - 5 days- Static - Biodentine - 5 days- Loading		-0.12436	0.9809
Biodentine - 5 days- Static - 9 BTO - 5 days- Static		-0.4745	0.9809
Biodentine - 5 days- Static - 9 BTO - 5 days- Loading	*	-1.81325	0.9809
Biodentine - 5 days- Static - Biodentine - 10 days- Static	*	-2.16426	0.9809
Biodentine - 5 days- Static - Biodentine - 10 days- Loading	*	-1.29598	0.9809
Biodentine - 5 days- Static - 9 BTO - 10 days- Static	*	-6.46715	0.9809
Biodentine - 5 days- Static - 9 BTO - 10 days- Loading	*	-4.46182	0.9809
Biodentine - 5 days- Loading - 9 BTO - 5 days- Static		-0.35014	0.9809
Biodentine - 5 days- Loading - 9 BTO - 5 days- Loading	*	-1.68889	0.9809
Biodentine - 5 days- Loading - Biodentine - 10 days- Static	*	-2.0399	0.9809
Biodentine - 5 days- Loading - Biodentine - 10 days- Loading	*	-1.17162	0.9809
Biodentine - 5 days- Loading - 9 BTO - 10 days- Static	*	-6.34279	0.9809
Biodentine - 5 days- Loading - 9 BTO - 10 days- Loading	*	-4.33746	0.9809
9 BTO - 5 days- Static - 9 BTO - 5 days- Loading	*	-1.33875	0.9809
9 BTO - 5 days- Static - Biodentine - 10 days- Static	*	-1.68976	0.9809
9 BTO - 5 days- Static - Biodentine - 10 days- Loading		-0.821475	0.9809
9 BTO - 5 days- Static - 9 BTO - 10 days- Static	*	-5.99265	0.9809
9 BTO - 5 days- Static - 9 BTO - 10 days- Loading	*	-3.98732	0.9809
9 BTO - 5 days- Loading - Biodentine - 10 days- Static		-0.351015	0.9809
9 BTO - 5 days- Loading - Biodentine - 10 days- Loading		0.517273	0.9809
9 BTO - 5 days- Loading - 9 BTO - 10 days- Static	*	-4.6539	0.9809
9 BTO - 5 days- Loading - 9 BTO - 10 days- Loading	*	-2.64857	0.9809
Biodentine - 10 days- Static - Biodentine - 10 days- Loading		0.868287	0.9809
Biodentine - 10 days- Static - 9 BTO - 10 days- Static	*	-4.30289	0.9809
Biodentine - 10 days- Static - 9 BTO - 10 days- Loading	*	-2.29756	0.9809
Biodentine - 10 days- Loading - 9 BTO - 10 days- Static	*	-5.17117	0.9809
Biodentine - 10 days- Loading - 9 BTO - 10 days- Loading	*	-3.16585	0.9809
9 BTO - 10 days- Static - 9 BTO - 10 days- Loading	*	2.00533	0.9809

* denotes a statistically significant difference.

- ***Gene Expression: DMP1***

ANOVA Table

Source	*Sum of Squares*	*Df*	*Mean Square*	*F-Ratio*	*P-Value*
Between groups	21.9673	7	3.13818	7.65	0.0001
Within groups	9.83884	24	0.409952		
Total (Corr.)	31.8061	31			

Contrast	*Sig.*	*Difference*	*+/- Limits*
Biodentine - 5 days- Static - Biodentine - 5 days- Loading		-0.423705	0.934417
Biodentine - 5 days- Static - 9 BTO - 5 days- Static		0.333015	0.934417
Biodentine - 5 days- Static - 9 BTO - 5 days- Loading	*	-1.40163	0.934417
Biodentine - 5 days- Static - Biodentine - 10 days- Static		0.501415	0.934417
Biodentine - 5 days- Static - Biodentine - 10 days- Loading		0.228945	0.934417
Biodentine - 5 days- Static - 9 BTO - 10 days- Static	*	-1.09807	0.934417
Biodentine - 5 days- Static - 9 BTO - 10 days- Loading	*	-1.85909	0.934417
Biodentine - 5 days- Loading - 9 BTO - 5 days- Static		0.75672	0.934417
Biodentine - 5 days- Loading - 9 BTO - 5 days- Loading	*	-0.977922	0.934417
Biodentine - 5 days- Loading - Biodentine - 10 days- Static		0.92512	0.934417
Biodentine - 5 days- Loading - Biodentine - 10 days- Loading		0.65265	0.934417
Biodentine - 5 days- Loading - 9 BTO - 10 days- Static		-0.674368	0.934417
Biodentine - 5 days- Loading - 9 BTO - 10 days- Loading	*	-1.43538	0.934417
9 BTO - 5 days- Static - 9 BTO - 5 days- Loading	*	-1.73464	0.934417
9 BTO - 5 days- Static - Biodentine - 10 days- Static		0.1684	0.934417
9 BTO - 5 days- Static - Biodentine - 10 days- Loading		-0.10407	0.934417

9 BTO - 5 days- Static - 9 BTO - 10 days- Static	*	-1.43109	0.934417
9 BTO - 5 days- Static - 9 BTO - 10 days- Loading	*	-2.19211	0.934417
9 BTO - 5 days- Loading - Biodentine - 10 days- Static	*	1.90304	0.934417
9 BTO - 5 days- Loading - Biodentine - 10 days- Loading	*	1.63057	0.934417
9 BTO - 5 days- Loading - 9 BTO - 10 days- Static		0.303555	0.934417
9 BTO - 5 days- Loading - 9 BTO - 10 days- Loading		-0.457463	0.934417
Biodentine - 10 days- Static - Biodentine - 10 days- Loading		-0.27247	0.934417
Biodentine - 10 days- Static - 9 BTO - 10 days- Static	*	-1.59949	0.934417
Biodentine - 10 days- Static - 9 BTO - 10 days- Loading	*	-2.3605	0.934417
Biodentine - 10 days- Loading - 9 BTO - 10 days- Static	*	-1.32702	0.934417
Biodentine - 10 days- Loading - 9 BTO - 10 days- Loading	*	-2.08804	0.934417
9 BTO - 10 days- Static - 9 BTO - 10 days- Loading		-0.761017	0.934417

* denotes a statistically significant difference.
